# Instant velocity and consistency of emitted cloud change by the different levels of canister filling with Metered Dose Inhalers (MDIs), but not with Soft Mist Inhalers (SMIs): a bench study

**DOI:** 10.1186/s40248-017-0096-1

**Published:** 2017-05-29

**Authors:** Roberto W. Dal Negro, Pietro Longo, Orestepaolo Villanis Ziani, Luca Bonadiman, Paola Turco

**Affiliations:** 1 National Centre for Respiratory Pharmacoeconomics & Pharmacoepidemiology – CESFAR, Verona, Italy; 2 Research & Clinical Governance, Verona, Italy

**Keywords:** Dose consistency, Instant velocity, MDIs, SMI, Variability

## Abstract

**Background:**

Inhalation is the preferred route for respiratory drug delivery, but several factors contribute to the variability of the respirable dose fraction. Instant velocity and the dynamic characteristics of the droplet cloud represent crucial factors. Aim was to measure and compare the instant velocity and the consistency of emitted cloud from five different MDIs (A - Salbutamol sulphate 100mcg, GSK; B - Salbutamol sulphate 100mcg, Valeas; C - Salmeterol xinafoate/Fluticasone propionate 25/125mcg, GSK; D - Formoterol fumarate/Bechlomethasone propionate 6/100mcg, Chiesi; E - Formoterol fumarate/Fluticasone dipropionate 5/125mcg, Mundipharma) and one SMI (Tiotropium bromide 5mcg, Boehringer Ingelheim), at different distance from the nozzle and canister filling.

**Methods:**

Measurements were made at 90, 50, and 10% of canister filling, and at 5, 10, and 20 cm from the nozzle, for a total of 972 puffs. A high speed video photography protocol was adopted and high speed cameras (1.200 frames/sec.) were used. Data were acquired by means of specialized softwares. Temperature, humidity, and vibrations occurrence were strictly controlled during measurements. Statistics: Anova and *p* < 0.05 were accepted as the minimum significance level.

**Results:**

MDIs generated different Instant velocities: MDI B generated the highest, while MDI A the lowest. As expected, velocity decreased in proportion to the distance from the nozzle. Except with MDI C, instant velocity decreased significantly over the first 50% of canister emptying, but dropped by >33% at 90% of emptying with all other MDIs (*p* < 0–037; *p* < 0.001; *p* < 0.005, and *p* < 0.001, respectively). Instant velocity was extremely lower (*p* < 0.001) and constant for all levels of canister filling (*p* = ns) with SMI. All MDIs had a very fast jet phase, ranging 0.01–0.03 s at 10 cm, and 0.03–0.05 s at 20 cm from the nozzle, without any significant difference from each other (*p* = ns). MDIs generated a cloud similarly tight (*p* = ns) at 10 and 20 cm from the nozzle, while it was extremely wider and constant with the SMI (*p* = 0–001). Also the cloud turbulence was minimized during the SMI emission.

**Discussion and Conclusions:**

MDIs are characterized by a substantial variability in both their instant velocity and consistency of the emitted cloud at different levels of canister filling. SMI generates a much slower soft mist cloud which is constantly homogeneous and independent of canister emptying. These peculiarities assessed at bench are suggesting a higher dose consistency and a much more effective therapeutic performance also in real life.

## Background

Inhalation is the preferred route for respiratory drug delivery since long ago. It allows the inhaled drug to target the bronchial tree directly with a lower dose, a quick onset of action, and a better therapeutic index [1].

Treatment of Bronchial Asthma (BA) and Chronic Obstructive Pulmonary Disease (COPD) stems from the use of several kinds of inhalers, which actually represent irreplaceable instruments for the effective therapeutic management of these pathological conditions, in both acute (i.e.: used as needed) and chronic (i.e.: for maintenance use) setting [2].

Dry Powder Inhalers (DPIs), Metered Dose Inhalers (MDIs) and Soft Mist Inhalers (SMIs) are widely used even if benefits to patients are strictly related to their usability [3–6]. Aerosol characteristics can further influence the effectiveness of airway treatment [7–9], being reproducibility of the emitted dose (such as the capability to consent the delivery of the same drug amount also in terms of its respirable fraction) one of the basic and indispensable characteristics for inhalers [2, 10].

Several factors contribute to the variability of the respirable fraction assumed from inhalers [11]. In particular, the instant velocity of the emitted dose from MDIs, together to the dynamic characteristics of their droplet cloud, can play a crucial role in causing such a variability [2–10]. Moreover, the instant velocity of emission can also change due to both the progressive emptying of the canister and the progressive depletion of the propellant [11].

Differently from MDIs, SMIs do not contain any propellant and their dose delivery is only driven by mechanical forces which force a metered dose of drug solution through a unique nozzle, thus producing two fine jets of liquid that converge at a pre-set angle. The collision of these two jets generates their typical soft mist [12, 13]. These characteristics are supposed to support a peculiar pattern of emission which should facilitate the effectiveness of drug inhalation [14, 15].

Nevertheless, quantitative studies aimed to compare MDIs and SMIs from this point of view are still episodic in real life [16–18].

### Aim

Aim of the study was to measure and compare the instant velocity and the consistency of emitted cloud from five different MDIs and one SMI, at different distances from the nozzle and at different levels of canister filling.

## Methods

Five MDIs were compared to the only SMI presently available on the market (such as: Tiotropium bromide 5mcg, Boehringer Ingelheim). They were made undistinguishable from each other before the measurements, and they were marked with sequential letters (A-E). Two MDIs were only containing salbutamol sulphate ((A - Salbutamol sulphate 100mcg, GSK and B - Salbutamol sulphate 100mcg, Valeas, respectively), while the other three a LABA/ICS combination (such as: C - Salmeterol xinafoate/Fluticasone propionate 25/125mcg, GSK; D - Formoterol fumarate/ Bechlomethasone propionate 6/100mcg, Chiesi, and E - Formoterol fumarate/ Fluticasone dipropionate 5/125mcg, Mundipharma, respectively). All devices were commercially available.

Six puffs from three canisters belonging to three different batches were measured for each kind of device. Measurements were carried out at 90, 50, and 10% of each canister filling, and at 5, 10, and 20 cm from the nozzle, for a total of 972 puffs.

A high speed video photography system was adopted and high speed cameras (1.200 frames/s.) were used (Casio Exilim PRO EX-F1; Shibuya, Tokio, Japan). Data were acquired by means of specialized softwares usually adopted for telemetric measurements (BioMOvie Tech; Aosta, Italy). All measurements were performed by expert engineers unaware of the devices’ content in a dark black room, where the light was optimized in order to maximize the contrast of the clouds generated. All procedures were conducted in the absence of any turbulence due to external factors, at a standard pressure (800 m above see level), and at a temperature of 18 ° C. During measurements, environmental conditions were strictly controlled in terms of temperature, humidity, and vibrations occurrence. The dose emission from each canister was electronically activated via a remote control. The rubber lever which caused the start of the emission was activated by a switch which also started the video recording on the computer hard disk.

Parameters collected were: the instant velocity (in m/s) measured at 5, 10, and 20 cm from the start of emission; the perimeter of the clouds emitted (in cm) calculated on thirteen points, and the corresponding areas (in cm^2^) at 10 and 20 cm from the nozzle.

Moreover, as concerning the uniformity of the cloud produced by each device, the portions of the emission characterized by high turbulence will appear in bright colours, while in dark colours those with the lowest turbulence of flow. A dynamic diagram also indicates the percentage of variation over all the emission phases. According to the colorimeter changes, more homogeneously dark the cloud, more consistent and less turbulent the emitted dose will be.

### Statistics

ANOVA for multiple comparisons of means ± sd was used, and *p* < 0.05 was accepted as the minimum level of significance.

## Results

### Instant velocity

Means ± sd of instant velocity calculated for each inhaler at different distances from the nozzle and at different filling levels of the canister are reported in Table 1, together with the significance level of comparisons. As expected, instant velocity was generally decreasing significantly in proportion to the distance from the nozzle with all inhalers (*p* < 0.001).

**Table 1:** Means (sd) of emitted dose velocity for each MDI and for the SMI, at different canister filling and distance from the nozzle. Anova for comparisons

		5 cm	10 cm	20 cm	Anova p
MDI A	90%	35.5 (7.9)	23.1 (1.6)	12.5 (1.5)	0.001
	50%	30.2 (10.0)	21.8 (2.4)	7.2 (6.4)	0.001
	10%	27.5 (9.7)	21.7 (2.3)	3.9 (6.2)	0.001
Anova p		<0.037	ns	<0.05	
MDI B	90%	63.1 (9.7)^a^	51.3 (5.7)	32.1 (2.4)	0.001
	50%	60.0 (9.3)^a^	51.7 (11.5)	31.4 (4.5)	0.001
	10%	42.3 (6.4)	33.3 (1.5%)	18.4 (0–9)	0.001
Anova p		<0.001	<0.001	<0.001	
MDI C	90%	47.4 (7.7)	28.1 (2.5)	17.9 (2.4)	0.001
	50%	40.5 (7.4)	25.5 (1.7)	16.7 (2.4)	0.001
	10%	37.7 (4.8)	27.9 (3.5)	17.8 (2.1)	0.001
Anova p		ns	ns	ns	
MDI D	90%	40.7 (11.7)	22.2 (1.4)	12.2 (0.8)	0.001
	50%	36.4 (6.9)	20.1 (2.0)	11.7 (1.1)	0.001
	10%	31.3 (3.7)	19.3 (1.6)	10.7 (1.2)	0.001
Anova p		<0.005	ns	<0.05	
MDI E	90%	45.0 (4.1)	32.0 (3.3)	17.2 (1.4)	0.001
	50%	41.4 (7.1)	29.3 (5.0)	16.1 (1.5)	0.001
	10%	31.2 (9.7)	21.7 (1.7)	12.9 (2.7)	0.001
Anova p		<0.001	<0.01	<0.02	
SMI	90%	5.7 (0.5)^b^	3.6 (0.5)	1.8 (0.4)	0.001
	50%	5.2 (1.6)^b^	3.5 (0.9)	1.8 (0.5)	0.001
	10%	5.0 (1.3)^b^	3.3 (1.1)	1.6 (0.7)	0.001
Anova p		ns	ns	ns	

^a^MDI A generated the highest value for instant velocity at 90% and 50% of canister filling (*p* < 0.001)

^b^SMI generated the lowest value for instant velocity at 90%, 50%, and 10% of canister filling (all *p* < 0.001)

Mean instant velocity was substantially different with each MDI, and variability confirmed by the large differences in corresponding value distributions (such as, sd). In particular, at 5 cm from the nozzle, MDI B generated the highest velocity (*p* < 0.001), while MDI A the lowest at 10 and also at 20 cm from the nozzle (*p* < 0.001) (Table 1).

With all MDIs, except MDI C, instant velocity decreased over the first 50% of canister emptying by 10–15%, and clearly dropped by 33% at 90% of emptying (*p* < 0–037; *p* < 0.001; *p* < 0.005, and *p* < 0.001, respectively). MDI A showed the highest decrease over the first 50% of canister emptying (such as, −15.0%), while MDI B and E proved the highest drop at 90% of emptying (such as, −33.0 and −30.7%, respectively) (Table 1).

In the case of SMI, instant velocity was extremely lower at 5 cm from the nozzle, and extremely constant for each level of canister emptying (*p* = ns). In particular, starting from 5.7 m/s at 5 cm, instant velocity still was 5.0 m/s at 20 cm from the nozzle, such as highly stable (Table 1).

### The jet and the cloud phase of emission

Data concerning the jet and the cloud phase of emission from each inhaler device are summarized in Table 2. Means ± sd of the time (in sec) needed to reach a distance of 10 and 20 cm from the nozzle are reported together to the means ± sd of the corresponding areas (in cm^2^) and perimeters (in cm) of the cloud, and the significance level of statistical comparisons.

**Table 2:** Means ± sd of time (sec.); area (cm^2^), and perimeter (cm) of the clouds calculated for each device at 10 and 20 cm from the nozzle. ANOVA for comparisons

	10 cm			20 cm		
	t sec	Area cm^2^	Perimeter cm	t sec	Area cm^2^	Perimeter cm
MDI A	0.011 (0.001)	13.71 (2.13)	20.54 (0.53)	0.05 (0.019)	71.10 (2.81)	45.05 (1.93)
MDI B	0.012 (0.01)	9.31^a^ (0.56)	19.19^a^ (0.55)	0.053 (0.01)	64.43 (2.50)	41.68 (1.30)
MDI C	0.011 (0.001)	16.72 (8.18)	21.87 (1.63)	0.03 (0.01)	52.42 (6.72)	43.1 (1.87)
MDI D	0.035 (0.021)	18.53 (4.61)	23.22 (1.33)	0.03 (0.01)	75.35 (2.41)	48.88 (2.84)
MDI E	0.03 (0.04)	20.48 (1.71)	24.24 (2.79)	0.05 (0.04)	59.46 (2.19)	47.01 (1.27)
SMI	0.29^b^ (0.04)	66.20^b^ (8.94)	38.68^b^ (11.14)	0.83^b^ (0.39)	130.08^b^ (5.71)	68.35^b^ (10.71)

^a^MDI B generated the tightest area and perimeter at 10 cm from the nozzle (ANOVA *p* < 0.001)

^b^SMI generated the widest areas and perimeters at 10 and 20 cm from the nozzle, with the slowest jet and cloud phase (ANOVA, all 0.001)

In particular, all MDIs showed a very fast jet phase, ranging 0.01-0.03 s at 10 cm, and 0.03-0.05 s at 20 cm from the nozzle, without any significant difference from each other (*p* = ns). As concerning the dynamic dimension and distribution of the cloud emitted, the tightest area and perimeter of the cloud were calculated for MDI B at 10 cm from the nozzle (*p* < 0.001), followed by MDI A and C (Table 2). All MDIs had comparable values at 20 cm from the nozzle (*p* = ns) (Table 2).

### The morphological characteristics of the emission cloud

The time needed to reach the 10 cm distance from the SMI nozzle was extremely longer (*p* < 0.001), by a 10/1 ratio when compared to that of MDIs (Table 2). Moreover, the dynamic dimension of the cloud was extremely wider at 10 and at 20 cm from the nozzle, thus showing a much slower, homogeneous and stable emission of the dose (*p* < 0.001) (Table 2).

### Uniformity of the cloud

**Fig. 1:**
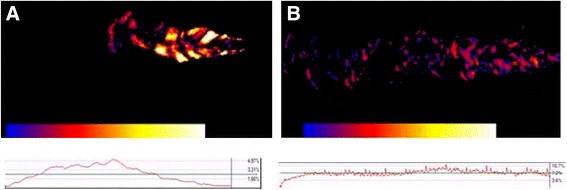
Colorimetric difference in the turbulence pattern obtained with MDI A (**a**) and SMI (**b**). The spots of *bright colours* indicates the sites of highest turbulence during the cloud emission, and their distribution. *Dark colours* indicate the absence of turbulence and the homogeneity of cloud emission. The variability of the *red diagram* trend indicates the corresponding dynamic stability or instability during all emission phases

**Fig. 2:**
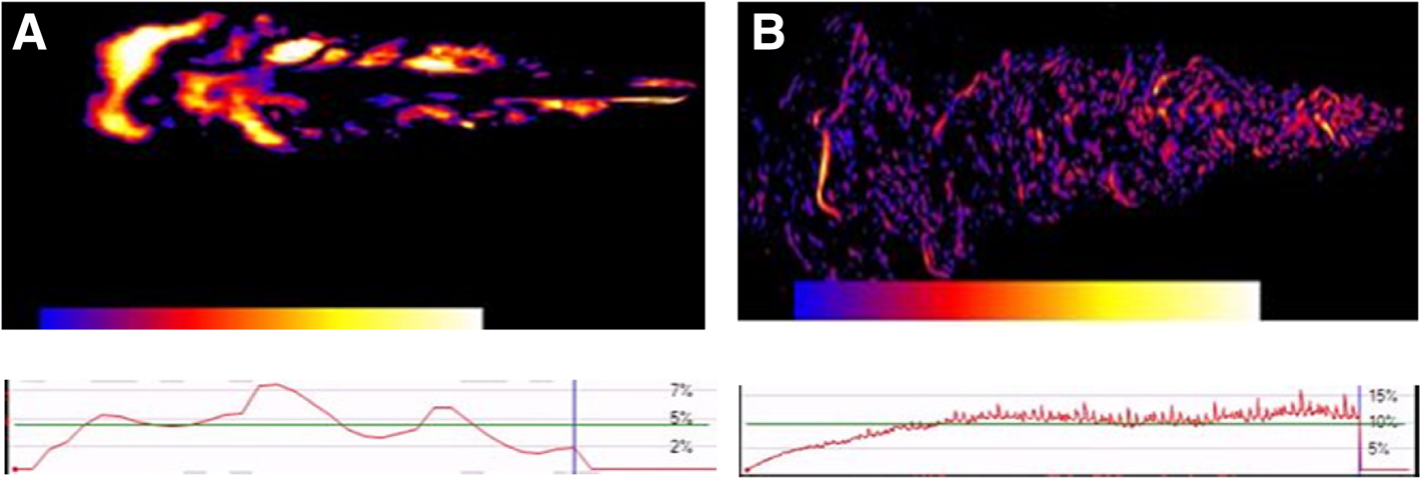
Colorimetric difference in the pattern of turbulence obtained with MDI B (**a**) and SMI (**b**). The spots of *bright colours* indicates the sites of highest turbulence during the cloud emission, and their distribution. *Dark colours* indicate the absence of turbulence and the homogeneity of cloud emission. The variability of the *red diagram* trend indicates the corresponding dynamic stability or instability during all the emission phases

The cloud turbulence was also minimized during the SMI emission. From this point of view, two examples of the difference between the flow turbulence generated by the MDI and the SMI are reported in Figs. 1 and 2, where the corresponding dynamic diagrams of the % variation from the start over all the emission phases are easily visible.

## Discussion

MDIs (Metered Dose Inhalers) still are the cheapest and the most widely prescribed devices in clinical practice, even if their use is frequently biased by several limitations, such as the high dependency of the patient’s cognition status; the patient’s physical limitations; the insufficient patient’s coordination between the device actuation and the required inspiratory flow. Due to these reasons, their use has to be frequently implemented with spacer devices in clinical practice [2–10, 16].

As well known, the performance of MDIs is characterized by a jet phase which is followed by a cloud phase [16]. MDIs suboptimal use mostly depends on the high velocity of these two phases of drug emission from the canister (i.e. even > 80 km/h from the nozzle), which can frequently lead to a quite poor therapeutic performance in real life [11]. Actually, droplet velocity can substantially affect the respirable fraction of the drug to inhale. It can be very poor in some circumstances [11, 17], as a large amount of the delivered drug is absorbed from the oro-pharingeal and/or gastric mucosa, and contributes to a relevant systemic bioavailability [19].

The performances of most used inhalers had been compared in a limited number of bench studies [16–18, 20, 21]. Data of the present study prove that a substantial variability in instant velocity of drug emission can occur with MDIs also in a strictly controlled experimental model, independently of the patient’s role. In particular, when compared to others, some MDIs are characterized by significantly higher velocity during either their jet and cloud phase of emission. Data on instant velocity obtained in the present bench study are in agreement with those reported in a recent review concerning the aerodynamic characteristics of inhaler devices, and in particular of MDIs and fixed combinations in asthma [21], even though, unfortunately, in this review the instant spray speed was not reported for all the same MDIs investigated in the present study.

Furthermore, only the velocity slow down obtained beyond 10 cm from the nozzle would consent a duration of drug delivery which is presumably fitting to an effective drug inhalation with such devices. To note that this distance from the mouth would be very difficult to be maintained by patients in real life without the aid of a spacer device, which is used in order to slow down all the phases of drug emission. In general, unless in the range 50–90% of canister emptying, instant velocity from MDIs still proves too high for allowing a “closed mouth technique” of inhalation to all kinds of patients in real life.

On the other hand, if the distance from the nozzle has been recognized to represent one of the most crucial points since long ago [19, 20], the homogeneity of the cloud emitted (such as the corresponding perimeter and area) had also been confirmed as able to affect the effectiveness of emission itself [11, 13, 16, 17].

A further crucial cause of variability in MDIs performance is represented by the substantial changes registered in instant emission velocity when calculated at different levels of canister filling. Consequently, the corresponding emitted dose can be easily supposed to change progressively during the canister life, and in proportion to its progressive emptying. This phenomenon represents a real limitation in MDIs reproducibility because patients, unaware of this phenomenon, will assume a dose of the drug(s) which is substantially different and progressively lower when the canister is at the beginning, rather than at the middle, or towards the end of its lifespan duration.

The five different MDIs tested in the present study also showed a clear inter-variability in their instant velocities of drug emission, likely due to their different construction criteria. From this point of view, while the instant velocity of MDIs named C, D, and E, such as those inhalers containing a β_2_ adrenergic/steroid combination, were more or less comparable, the performances resulted quite different and variable in the case of MDIs A and B, such as those inhalers only containing salbutamol. In particular, these two latter devices, which are the most used in emergency situations, are afflicted by a high inter- and intra-variability for different canister filling and for different distances from the nozzle. In other words, they should not be regarded as interchangeable in clinical practice because the different dynamic patterns of their cloud emission can correspond to highly variable and dramatically unpredictable therapeutic performances. From this point of view, also the high turbulence registered during the MDIs emission can contribute to further affect the reproducibility of their emitted dose (Figs. 1 and 2).

In general, SMIs (Soft Mist Inhalers) did not result afflicted by these critical aspects. In particular, data of the present study proved that, when compared to those of MDIs, the instant velocity of drug emission from SMIs is dramatically lower (by a 10/1 ratio), and absolutely constant for any level of canister filling. Obviously, instant velocity tends to decrease in proportion to the distance from the nozzle also in this case, but the range of absolute values is dramatically smaller, and the pattern of the cloud proved much more homogeneous and consistent for all their emission phases, and not characterized by any significant turbulence. Moreover, even if the perimeter of the SMI cloud is larger than those produced by MDIs, no negative effects on drug deposition can be presumed thanks to the dramatically lower velocity of the emission, which allows the vast majority of patients to inhale the cloud effectively.

The characteristics of the cloud generated by the SMI proved absolutely different. In agreement with other studies [12, 13, 18], present results prove the extremely higher stability of cloud emission from the SMI devices, which likely contributes to explain their easier and more convenient use to the patient [22]. In particular, both the homogeneity and the consistency of the emitted cloud represent the peculiar characteristics of SMIs’ performance.

Whether the results of the present bench study support the principle that the difference and variability in instant velocities, cloud morphology, and consistency assessed for MDIs and the SMI can affect their effectiveness/efficacy, further studies aimed to investigate specifically the effects of these differences in clinical practice should be planned.

## Conclusions

The dynamic characteristics of the soft mist cloud assessed during the present bench study are suggesting a much more effective performance of SMIs when compared to that of MDIs. This peculiarity is mainly due to the slower jet emission and to the much more homogeneous composition of the droplet cloud generated.

## Abbreviations

BA: Bronchial Asthma
COPD: Chronic Obstructive Pulmonary Disease, MDIs, Metered Dose Inhalers
SMIs: Soft Mist Inhalers
